# Dietary Strategies to Improve Cardiovascular Health: Focus on Increasing High-Density Lipoprotein Functionality

**DOI:** 10.3389/fnut.2021.761170

**Published:** 2021-11-22

**Authors:** Julia T. Stadler, Gunther Marsche

**Affiliations:** Division of Pharmacology, Otto Loewi Research Center, Medical University of Graz, Graz, Austria

**Keywords:** Mediterranean diet, polyphenols, HDL composition, paraoxonase1, cholesterol efflux capacity

## Abstract

Cardiovascular disease is one of the leading causes of morbidity and mortality worldwide, with increasing incidence. A cornerstone of cardiovascular disease prevention is lifestyle modification through dietary changes to influence various risk factors such as obesity, hypertension and diabetes. The effects of diet on cardiovascular health are complex. Some dietary components and metabolites directly affect the composition and structure of high-density lipoproteins (HDL) and increase anti-inflammatory and vasoprotective properties. HDLs are composed of distinct subpopulations of particles of varying size and composition that have several dynamic and context-dependent functions. The identification of potential dietary components that improve HDL functionality is currently an important research goal. One of the best-studied diets for cardiovascular health is the Mediterranean diet, consisting of fish, olive oil, fruits, vegetables, whole grains, legumes/nuts, and moderate consumption of alcohol, most commonly red wine. The Mediterranean diet, especially when supplemented with extra virgin olive oil rich in phenolic compounds, has been shown to markedly improve metrics of HDL functionality and reduce the burden, or even prevent the development of cardiovascular disease. Particularly, the phenolic compounds of extra virgin olive oil seem to exert the significant positive effects on HDL function. Moreover, supplementation of anthocyanins as well as antioxidants such as lycopene or the omega−3 fatty acid eicosapentaenoic acid improve parameters of HDL function. In this review, we aim to highlight recent discoveries on beneficial dietary patterns as well as nutritional components and their effects on cardiovascular health, focusing on HDL function.

## Introduction

Cardiovascular disease (CVD) is one of the leading causes of death worldwide and the numbers are on the rise. Data obtained in 2018 indicate that CVD is responsible for more deaths than cancer and chronic lung disease combined (1). Risk factors for CVD comprise age, sex, hypertension, dyslipidemia, and diabetes. However, the likelihood of developing CVD is also increased by various health behaviors such as smoking and tobacco use, physical inactivity, obesity, and most importantly, nutrition. An unhealthy diet, which contributes to disease probability is characterized by increased consumption of processed foods, unhealthy fats, sodium, and added sugars (2–4).

In contrast, results of dietary intervention studies suggest that various foods and healthy dietary patterns, such as the Mediterranean diet, are associated with a markedly lower risk of CVD (5).

Atherosclerosis is an inflammatory disease that underlies a major part of the incidence and mortality of CVD. The inflammatory state promotes the accumulation of extracellular lipids or macrophage foam cells in the vessel wall, leading to atherosclerotic lesions. A poor diet and physical inactivity are risk factors for the disease, but lifestyle changes can prevent the development of atherosclerosis, due to several factors, such as reducing oxidative stress and decreasing the release of pro-inflammatory cytokines (6, 7).

Based on the close relationship between HDL-cholesterol (HDL-C) levels and CVD, efforts have long been made to reduce the risk of the disease by increasing plasma HDL-C levels (8). However, to this point therapeutics to increase HDL-C levels have failed, indicating that simply raising the quantity of HDL-C does not protect from CVD (9, 10). The negative result of HDL-C raising strategies may be partly explained by the recently demonstrated U-shaped association between HDL-C and CVD, with both extreme high- and low HDL-C concentrations associated with increased mortality, indicating that plasma levels of HDL-C do not accurately reflect the atheroprotective potential of HDL (11, 12).

It has to be noted that there is no clear explanation for the “paradoxical” association of very high HDL-C and increased mortality. One hypothesis is that in individuals with extremely high HDL-C, the functional properties of HDL are altered such that HDL no longer functions normally. Given the heterogeneity of HDL particles in terms of structure, size, lipidomic/proteomic composition, and metabolism, steady-state HDL-C levels suffer from the limitations imposed by their mass-based and static measurement. As a snapshot of the steady-state plasma cholesterol levels, HDL-C levels do not provide direct information on the rate of cholesterol efflux from vascular macrophages to the liver, which is influenced by many factors beyond the mass of HDL-C alone. Therefore, circulating HDL-C concentrations do not provide information about the structure and composition of HDL and anti-inflammatory, antioxidant, antithrombotic, and endothelial function-promoting activities of HDL (13–15), although there is increasing evidence of the clinical importance of these pleiotropic functions (16). Therefore, current research strategies focus on improving the atheroprotective functions of HDL.

Recent studies have shown that several dietary strategies and various nutritional components can affect levels of HDL-C and improve/affect some of the atheroprotective functions of HDL. In this review, we summarize established and novel approaches found in literature on the effects of several dietary approaches to influence HDL composition and function, with a particular focus on nutritional phenolic compounds and the Mediterranean diet.

## HDL Metabolism

**Figure 1:**
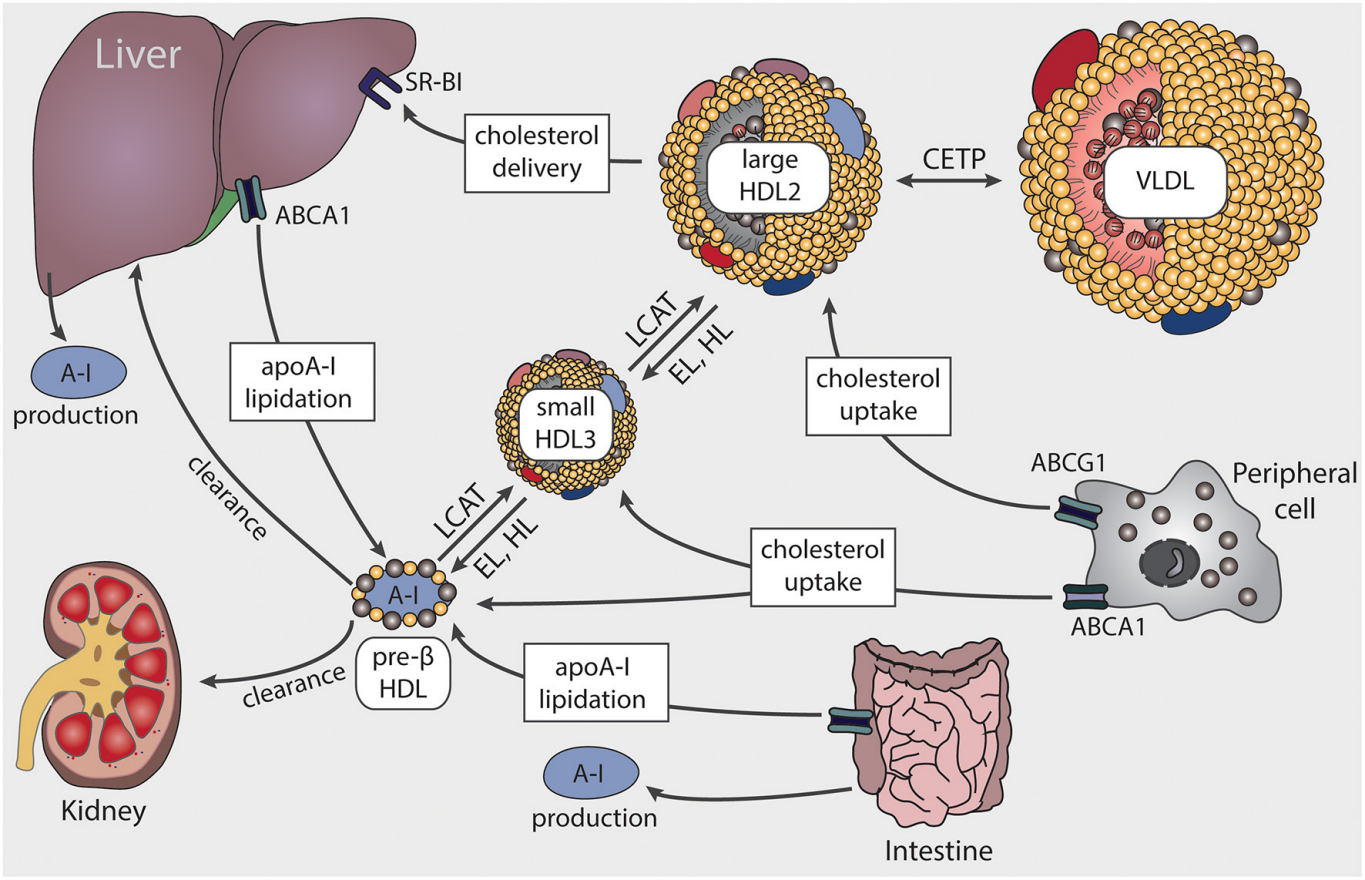
Schematic representation of HDL biosynthesis and maturation. HDL biosynthesis starts with the production and secretion of apolipoprotein A-I (apoA-I) by the liver and intestine. Lipid-poor apoA-I interacts with ATP-binding cassette A1 (ABCA1) to acquire lipids, resulting in pre-β HDL formation. Through lecithin-cholesterol-acyl transferase (LCAT), the ingested free cholesterol on the surface of HDL is esterified to cholesteryl-ester forming larger particles. ABCA1 preferentially interacts with pre-β HDL or small HDL3 particles, while ATP binding cassette G1 (ABCG1) stimulates cholesterol transfer to larger HDL2 particles. Cholesterol is delivered to the liver via scavenger receptor BI (SR-BI) or transferred to very low-density lipoproteins (VLDL) by cholesteryl-ester transfer protein (CETP). HDL-associated triglycerides and phospholipids are mainly hydrolyzed by endothelial lipase (EL) and hepatic lipase (HL).

The first step in the formation of HDL is the production and secretion of the major HDL apolipoprotein, apoA-I, predominantly from the liver and the intestine (Figure 1) (17). After secretion, lipid-poor apoA-I interacts with ATP-binding cassette A1 (ABCA1) to acquire cholesterol and phospholipids from cellular lipid pools, which leads to the formation of nascent HDL particles. Cholesterol efflux from peripheral cells results in HDL particles becoming progressively larger and enriched in cholesterol. The acquired cholesterol on the surface of HDL is subsequently converted by the enzyme lecithin-cholesterol acyltransferase (LCAT) into cholesteryl-esters, which form the core of HDL particles (18). ABCA1 preferentially stimulates cholesterol efflux to pre-β HDL and small HDL3 particles, while ATP binding cassette G1 (ABCG1) interacts with large HDL2 particles (19, 20). Further uptake of lipids by HDL occurs via transfer of surface components of triglyceride-rich lipoproteins, during lipolysis by lipoprotein lipase (21).

Clearance of HDL cholesteryl-esters can occur *via* two different routes. First, the cholesterol content of HDL can be taken up selectively by scavenger receptor B1 (SR-BI) from the liver or steroidogenic tissues. Alternatively, cholesteryl-ester clearance can be mediated by cholesteryl-ester transfer protein (CETP), which transfers cholesteryl-ester from HDL to triglyceride-rich lipoproteins, in exchange for triglycerides. The triglyceride-enriched HDL particles are then more susceptible to lipolysis and rapidly catabolized by hepatic or endothelial lipase. Clearance of apoA-I then occurs in the kidney and liver (22). The interplay of the various apolipoproteins, lipid transfer proteins, enzymes, and surface receptors result in HDL particles of distinct sizes and functionality.

## HDL Structure, Composition, and Function

Of particular interest, certain diets and dietary components affect HDL composition, especially lipid components, but also the protein content of HDL can be affected.

HDL particles are very heterogeneous and differ in their size depending on their site of origin, proteomic and lipidomic composition and maturation stage. Approximately 70% of the total protein content of HDL accounts for apoA-I. This apolipoprotein acts as an activator of LCAT, interacts with cellular receptors and exerts several antiatherogenic activities (23). The second major apolipoprotein of HDL is apoA-II with 15–20% of the total protein amount. Other major HDL associated proteins are apoC-II, which serves as an activator of lipoprotein lipase, whereas apoC-III is an inhibitor. ApoE is a key functional apolipoprotein as well (24). Most circulating apoE is associated with triglyceride-rich lipoproteins, where it serves as a ligand for apoE/apoB receptors and facilitates binding of lipoproteins to cell surfaces. Other minor apolipoprotein components of HDL are apoM, apoA-IV, apoF, apoD, apoJ and apoH, apoO, and apoL-I.

Pre-β particles are the structurally simplest form of HDL. These particles are lipid-poor, monomeric or dimeric apoA-I molecules and account for about 5% of the apoA-I content in the circulation (25). Pre-β particles are discoidal in shape and have a molecular weight of ~70 kDa. Through their rapid uptake of cholesterol and phospholipids, pre-β particles are transformed into larger HDL subgroups. The small HDL3 particles have a density of 1.125–1.21 g/ml, are rich in proteins and have a molecular weight of ~175 kDa. The larger size of HDL2 particles is reflected by their increased lipid content. This subclass has a density range of 1.063–1.125 g/ml and a molecular weight of about 350 kDa. In terms of HDL functionality, HDL3 particles have been proposed as the more anti-atherogenic HDL subclass in the general population. The smaller and denser particles display potent cholesterol efflux capacity and possess high antioxidative and anti-inflammatory activities (15, 26). These differences in HDL functionality between the subclasses can be partially explained by their differential proteomic and lipidomic composition. Several proteins are preferentially present on HDL3 particles, such as PON1, apoA-II, and apoM (27). ApoM provides a hydrophobic binding pocket that allows sphingosine-1-phosphate (S1P) to bind (28), which also has shown higher abundance on the HDL3 subclass (29). The apoM/S1P complex exerts several anti-inflammatory and endothelium-protective activities, which seem to account for at least some of the antiatherogenic activities of HDL (30). Recent research further demonstrated that HDL3 produced by the intestine efficiently sequesters lipopolysaccharide (LPS) and thereby protects against liver inflammation (31). Enterically derived HDL3 is enriched in LPS-binding protein and masks LPS from detection by Toll-like receptor 4 (31).

Due to its influence on oxidative stress and inflammation, the activity of the HDL-associated enzyme paraoxonase 1 (PON1) has been investigated in several pathological conditions, including vascular diseases (32, 33), renal disease (34, 35), diabetes (36–39), and cancer (40). Importantly, it has been reported that PON1 activity can be modulated by implementing certain lifestyle habits and dietary patterns, which will be discussed in more detail.

PON1 has a wide range of substrates that can be hydrolyzed. PON1 is mainly expressed and secreted into circulation from the liver, but also to some extent in kidneys and colon (41). Due to its antioxidative capacity, it has been suggested as an important player in atheroprotection (42). PON1 is very unstable, therefore its association with HDL is important to ensure stabilization and to maintain serum enzyme activity (43, 44). PON1 was originally described to hydrolyze organophosphates such as paraoxon, a metabolite of the pesticide parathion (45). However, more recent studies have demonstrated that PON1 is further able to hydrolyze homocysteine thiolactone, which is a known risk factor for CVD and predictor for CVD mortality (46, 47). Therefore, PON1 is considered to be a protective factor against coronary artery disease (48). Additionally, purified PON1 protects both HDL and LDL from oxidative modifications caused by oxidized lipids (49–51). This ability of PON1, to inactivate the oxidized lipids was attributed to a specific cysteine residue at position 283. PON1-knockout mice show a higher susceptibility to endothelial dysfunction and atherosclerosis (52).

The HDL lipidome is largely composed of phospholipids (40–60%) and cholesteryl-esters (30–40%), while triglycerides (5–12%), and free cholesterol (5–10%) account for smaller proportions. Lipidomic analyses have identified over 200 different lipid species, which, together with the different protein components, are responsible for the high heterogeneity of HDL particles (29).The association of HDL subfractions with HDL function and cardiovascular risk is complex and incompletely understood. In the general population, smaller HDL particles have been shown to be more protective, whereas diameter, cholesterol- and triglyceride- content of very large HDL particles is associated with CAD risk (53). However, several chronic diseases are associated with profound alterations in HDL metabolism and function, caused by increased systemic oxidative stress and inflammation (12, 54). These conditions include obesity (55–57), chronic kidney disease (58, 59), liver disease (60, 61), diabetes (62–64), CVD (65, 66), but also allergic rhinitis (67) and skin diseases (68, 69). Compositional modifications and concomitant changes in parameters of HDL function may lead to development of pro-atherogenic characteristics and enhancement of the inflammatory state. HDL cholesterol efflux capacity is significantly influenced by both the concentration and the functionality of specific HDL particles participating in cell-cholesterol efflux. CAD patients have higher than normal preβ-1 concentrations with decreased functionality, and lower than normal large HDL particle concentrations (70). Concentrations of small HDL particles are sometimes even inversely correlated with cholesterol efflux capacity (71). This suggests a block in maturation of small HDL particles in inflammatory disease states and a complex interrelationship between the lipid-binding capacity of apoA-I and the functionality of HDL particles in disease (72).

### HDL-Functionality and Cardiovascular Health

**Figure 2:**
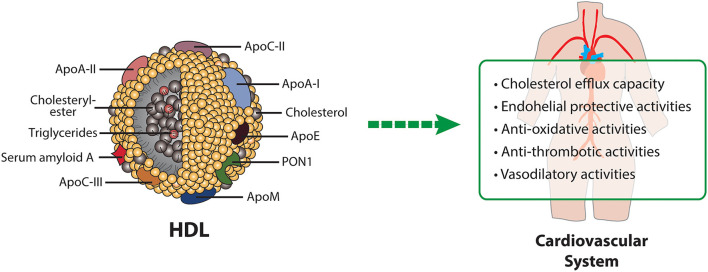
HDL composition and HDL-mediated protective mechanisms in cardiovascular disease. Apo, apolipoprotein; PON1, paraoxonase1.

HDL particles display several biological activities, which are involved in atheroprotection (Figure 2). The best studied activity of HDL is the ability to remove excess cholesterol from arterial wall cells and subsequent delivery to liver and steroidogenic organs. The first step of reverse cholesterol transport is commonly referred to as the cholesterol efflux capacity of HDL. Indeed, it was shown that this function has a strong inverse association with coronary artery disease, independent of HDL-C levels (73). Another important antiatherogenic function of HDL is endothelial protection. Vascular injury or pro-inflammatory cytokines induce the expression of several adhesion molecules on the endothelium, which attract leukocytes and allow transmigration into the intima. HDL reduces cytokine-triggered expression of adhesion molecules on endothelium, thereby inhibiting adhesion of monocytes to endothelial cells and having a protective effect on endothelium (74–76). This anti-inflammatory capacity, which can be measured in a cell based assay, is inversely associated with incidence of cardiovascular events in the general population (77). Furthermore, HDL is capable to reduce the expression of chemokines and chemokine receptors via nuclear factor B and peroxisome proliferator–activated receptor γ modulation (78). Moreover, HDL has been identified as an important mediator of endothelial progenitor cell mediated cell repair (79, 80). Specifically, HDL pre-incubated endothelial progenitor cells showed improved adhesion to human coronary artery endothelial cells and up-regulated β_2_-integrins, which play a unique role in endothelial progenitor cell adhesion (80). Moreover, after injection of recombinant HDL into a mouse model with inflammatory de-endothelialization, endothelial progenitor cell—mediated repair of the endothelium was enhanced (81). Furthermore, in patients with coronary artery disease, a correlation between HDL and circulating endothelial progenitor cells was observed (80). The vasodilatory activity of HDL is generally reflected by its ability to induce endothelial nitric oxide (NO) release, but also prostacyclin production (11, 82–85). HDL mediated activation of endothelial NO synthase is dependent on AMPK activation, which is in turn dependent on S1P-receptors and SR-BI (11).

In addition, HDL is thought to act atheroprotective by reducing oxidative stress. HDL protects other lipoproteins from oxidative damage by removing oxidized lipids caused by free radicals. Components of HDL, such as apoA-I or HDL-associated PON1 are involved in the reduction or hydrolyzation of oxidized lipids (86–91). Additional protective activities of HDL include its antithrombotic effects based on several mechanisms, such as reduced susceptibility of platelets to aggregation and reduced activation of the coagulation cascade (92). Platelet activation is prevented by HDL-induced upregulation of endothelial NO and prostacyclin synthesis (93) and downregulation of thromboxane A2 synthesis and platelet activating factor release (85).

## Dietary Strategies and HDL Function

### Mediterranean Diet

**Figure 3:**
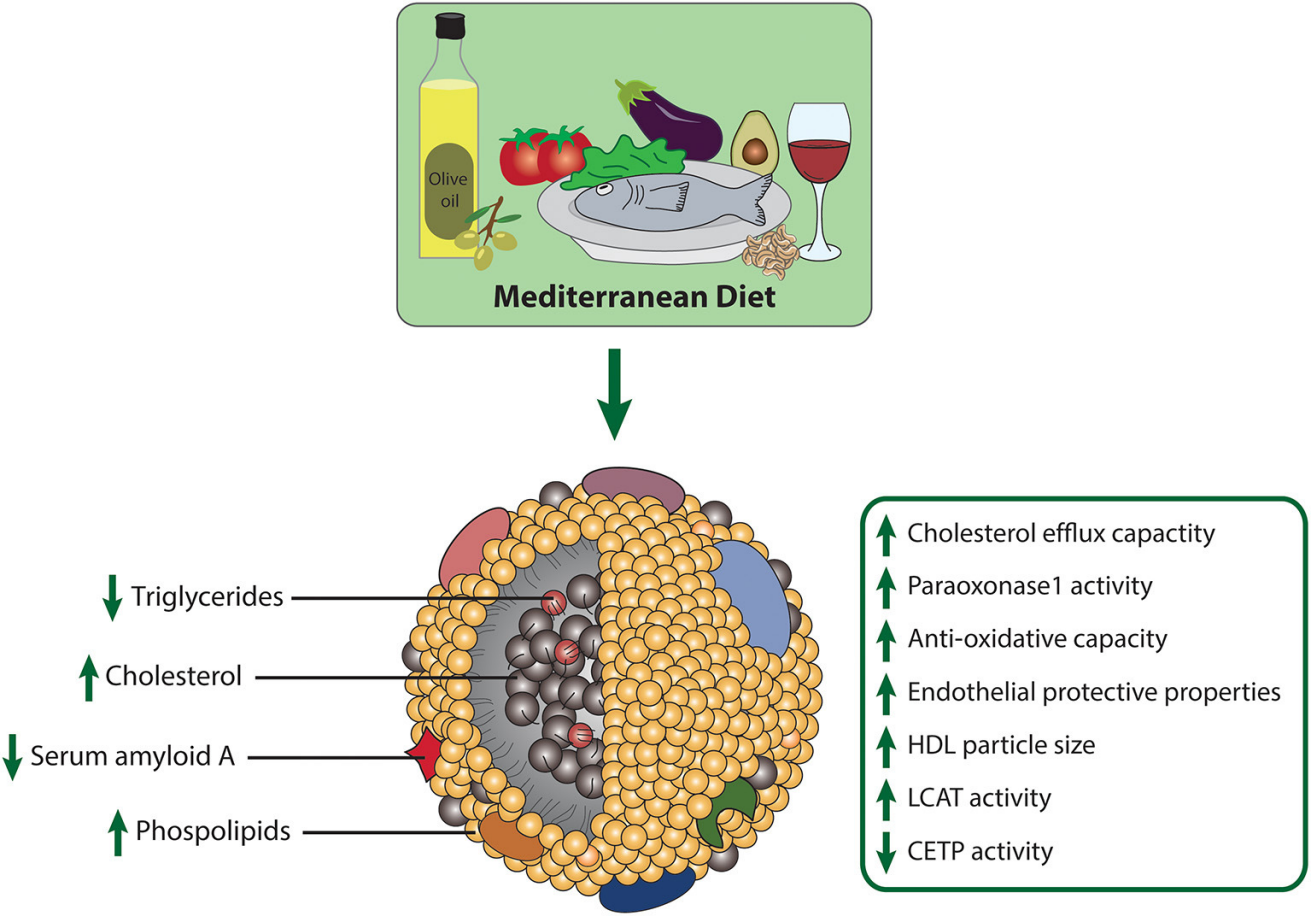
Effects of the Mediterranean diet on composition and metrics of HDL function.

The nutritional strategy known as the Mediterranean diet is becoming increasingly popular. The Mediterranean diet is characterized by high intake of extra virgin olive oil (EVOO), vegetables, nuts, legumes, whole grain products and fish, moderate consumption of alcohol, typically red wine, and low intake of red and processed meat, poultry and dairy products (94). Interest in the diet began in the 1950's when it was noted that heart disease was not as common in Mediterranean countries. Since then, numerous studies have confirmed that the Mediterranean diet helps to prevent heart disease and stroke (95). Notably, the Mediterranean diet is the only dietary pattern, which was shown to markedly improve HDL-functional parameters (Figure 3). The PREDIMED trial was one of the largest randomized controlled trials to explore the effects of the Mediterranean diet on cardiovascular disease prevention (5). In this study, 7,447 high-cardiovascular-risk patients were enrolled and assigned to one of three different diets: (1) Mediterranean diet supplemented with nuts, (2) Mediterranean diet supplemented with EVOO, (3) and a control diet with reduced fat intake. Lower incidence of cardiovascular events was observed in both of the groups supplemented with EVOO and nuts (5). In a random subsample of 296 participants of the PREDIMED trial, Hernáez et al. analyzed the effect of this diet on HDL functionality (96). After 1-year intervention, cholesterol efflux capacity of HDL was increased in both Mediterranean diet groups, compared to baseline levels (96). The authors suggested that the improvement in efflux capacity may be explained by increased HDL-related gene expression, changes in HDL-associated lipids and enhanced antioxidative capacity of HDL. Further analyses revealed that in the intervention group supplemented with EVOO, the ability of HDL to esterify cholesterol significantly increased, while activity of CETP decreased relative to baseline levels. LCAT is highly sensitive to oxidative modifications, therefore dietary consumption of antioxidative compounds may protect against oxidative inactivation (97). The arylesterase activity of PON1 did not change after the intervention; however, compared to the low-fat control diet, the activity was increased in the EVOO supplemented group. In addition, the ability of HDL to counteract LDL oxidation increased after EVOO intervention compared to baseline. Concerning compositional parameters of HDL, the authors found a reduced content of triglycerides after both Mediterranean diet interventions, compared to the low-fat control group. Further, the content of HDL surface phospholipids increased in the EVOO group, when compared to baseline and the control group. In this study, the dietary intervention had no effect on apoA-I, apoA-II, and apoC-II content of HDL (96). Further analyses of HDL functional parameters with the consumption of several food groups revealed that the decline in CETP activity was associated with legume and fish consumption (98). Moreover, EVOO intake and whole grain consumption was associated with increased cholesterol efflux capacity, while legume and fish intake was linked to increments of PON1 activity (98). The effect of EVOO on PON1 activity has been confirmed in other studies as well (99, 100). In patients with metabolic syndrome, a 12-week intervention with a Mediterranean diet and additional exercise markedly improved HDL cholesterol efflux capacity (101).

A 3 week intervention with polyphenol-rich olive oil improved cholesterol efflux capacity and increased HDL particle size compared to the control group receiving polyphenol-poor olive oil (102). Olive oil polyphenols increased HDL cholesterol efflux capacity and enhanced the anti-oxidative capacity of HDL through an increase in the olive oil phenolic compounds, such as hydroxytyrosol, glucoronate, and homovanillic acid sulfate. HDL-enrichment with these antioxidative metabolites is expected to provide protection against oxidative modifications (103). Olive oil polyphenols further increased HDL size and promoted a greater HDL stability, reflected by a triglyceride-poor core related to a more stable conformation of apoA-I and PON1 (99, 102–104).

More recently, the effect of EVOO intake on cholesterol efflux capacity of HDL in young and elderly study subjects was investigated. Of particular interest, cholesterol efflux capacity was lower in the elderly group, but returned to normal levels after 12-week of EVOO intervention (105). In addition, HDL subclass analyses showed lower levels of large HDL in the elderly group, which increased again after the intervention. Linear regression analyses showed a strong correlation of large HDL particles with cholesterol efflux. The age-related decrease in cholesterol efflux capacity was partly explained by the alteration in the distribution of HDL subclasses, which was modulated after the 12-week EVOO intervention (105).

Tomatoes are readily consumed as part of a Mediterranean diet, and a study of 39,000 women found that ingestion of seven or more servings of tomato-based products per week was associated with a 30% reduction in relative risk of CVD (106). The potential cardiovascular benefits of a tomato-rich diet may be attributed to their high lycopene content, especially as tomatoes account for up to 80% of dietary lycopene intake (107). In one study, HDL functionality was assessed following lycopene supplementation (70 lycopene/week) by monitoring the activities of PON1, CETP, LCAT, and serum amyloid A (SAA) content of HDL (108). After supplementation, lycopene content increased in HDL, and in parallel, PON1 and LCAT activities increased, whereas the content of pro-inflammatory HDL-associated SAA and CETP activity decreased. These results suggest that increased lycopene intake leads to beneficial changes in HDL metabolism, structure and function.

Fish or fish oils, rich in omega-3 fatty acids, are consumed as part of a Mediterranean diet and have been linked to a lower risk of CVD (109). Eicosapentaenoic acid (EPA) is an omega-3 fatty acid that has been shown to reduce levels of pro-atherogenic small dense LDL, remnant lipoprotein particles, and C-reactive protein in metabolic syndrome, presumably due to suppression of hepatic triglyceride production and degradation of CETP after supplementation (110). In patients with dyslipidemia, treatment with EPA (1,800 mg/day) has been shown to improve HDL function, enhancing HDL cholesterol efflux capacity and antioxidant and anti-inflammatory activities (111). EPA-enriched HDL inhibited cytokine-stimulated endothelial VCAM-1 expression and increased production of the anti-inflammatory EPA-derived metabolite resolvin E3 (112). Furthermore, *in vitro* studies revealed that EPA inhibits oxidation of HDL in a dose-dependent manner, which may contribute to the preservation of the antiatherogenic properties of HDL (113). In contrast, the omega-3 fatty acid docosahexaenoic acid (DHA −22:6; n-3) showed an initial antioxidative effect, but this was lost over time. However, comparison studies of EPA and DHA demonstrated that these fatty acids have distinct effects on plasma lipids, with DHA administration being more efficient in raising HDL-C, particularly the HDL2 subfraction and increasing LDL particle size (114–116). A recent study analyzed the effects of 8-week EPA and DHA supplementation on lipoprotein subfractions and HDL proteome in healthy and normolipidemic participants (117). The authors revealed that both fatty acids led to a reduction of VLDL-particle size and VLDL-particle number, suggesting a reduced hepatic VLDL production (118). Both EPA and DHA administration led to a reduction in medium sized HDL-particles and increased large HDL subfraction number. Of particular interest, proteomic analyses showed that supplementation with EPA-rich fish oil increased HDL apoM levels and decreased proteins involved in inflammation (117).

Similar to olive oil, nuts are enriched with mostly monounsaturated and polyunsaturated fatty acids and contain many vitamins and phytosterols (119). Especially walnuts are a rich source of α-linolenic acid and α-linoleic acids and have been shown to improve plasma lipid levels (120). Moreover, acute consumption of walnuts improves HDL cholesterol efflux capacity, while walnut oil demonstrated beneficial effects on endothelial function (121). In a randomized controlled trial of high-risk CVD patients, the effects of three isocaloric diets were investigated to examine whether the beneficial effects of walnuts on lipid/lipoprotein levels are attributable to their fatty acid content (122). Replacement of saturated fatty acids with unsaturated fats from walnuts or vegetable oils improved lipid/lipoprotein classes, including LDL-cholesterol, non-HDL cholesterol, and total cholesterol but did not affect HDL cholesterol efflux capacity (122).

Another characteristic of the Mediterranean diet is moderate alcohol consumption, usually at mealtimes and in the form of red wine. Consumption of alcoholic beverages is associated with increased plasma levels of HDL-C, phospholipids and apoA-I and a reduction in CETP activity (123–125). Moreover, alcohol is a consistent dietary factor that has shown a positive effect on cholesterol efflux capacity. Moderate intake of alcohol is associated with increased cholesterol efflux (126, 127), but also heavy alcohol intake was shown to enhance cholesterol efflux to HDL2 particles, concomitant with an increase of larger particles (125). The increased cholesterol efflux capacity might be due to the increase of HDL-phospholipids observed in alcohol consumers (128). Interestingly, a study compared the effects of beer, red wine, and spirituous (Dutch gin) consumption on cholesterol efflux and plasma cholesterol esterification-rate. All alcoholic beverages significantly increased cholesterol efflux, without any differences between groups, while plasma esterification rate showed a significant increase after beer and spirituous consumption (126). Of particular interest, 3-week consumption of these beverages also increased PON1 activity, which was strongly correlated to increased HDL-C and apoA-I (129).

Like nuts and olive oil, avocados are a nutrient-rich source of polyphenols and monounsaturated fatty acids (130). In a study comparing different cholesterol-lowering diets, supplementation with 136 g avocado per day for 5 weeks resulted in a reduction of LDL-C and non-HDL-C compared to baseline (131). In addition, the avocado diet reduced LDL particle number, small and dense LDL-C and improved the LDL/HDL ratio (131). The beneficial effect on LDL-C was greater in the avocado diet group than in a diet containing moderate fats and oleic acid oils. These results suggest additional beneficial effects of avocado consumption.

In conclusion, the Mediterranean effectively prevents cardiovascular disease, and the improvement of atheroprotective functions of HDL likely contributes to this. Especially EVOO consumption has been shown to be a potential therapeutic option to promote the cholesterol efflux capacity of HDL. In addition, the antioxidant compounds in EVOO, lycopene, but also fish-derived omega-3 fatty acids may protect HDL from oxidative changes, resulting in more stable and functional particles (103). Moreover, moderate alcohol consumption has a positive effect on HDL cholesterol efflux capacity and appears to increase PON-1 activity.

### Caloric Restriction and Intermittent Fasting

Reducing calorie intake, without malnutrition, are commonly implemented lifestyle interventions to lose weight or to improve general health. Of particular interest, caloric restriction, together with intermittent fasting, appear to be an effective dietary intervention to robustly enhance health and reduce age-associated parameters in several organisms (132). Human studies on caloric restriction in non-obese participants reported lower levels of oxidative stress, reduced fasting insulin levels as well as lower circulating levels of tumor necrosis factor alpha (133–135). Furthermore, caloric restriction caused a reduction in body weight as well as an improvement of cardiometabolic health parameters. Effects of caloric restriction on HDL composition and function have been investigated in a few small studies. In a study including 27 diabetic and obese patients, 16-weeks of a low-calorie diet (450 kcal/day) resulted in increased plasma apoA-I levels and markedly decreased plasma CETP concentration, but did not alter cholesterol efflux capacity of HDL (136). Another study evaluated the long-term effects of caloric restriction on risk factors of atherosclerosis (137). Eighteen participants who had been on caloric restriction for an average of 6 years were compared with 18 age-matched individuals who followed a typical American diet. In the caloric restriction group, several risk factors for atherosclerosis were lower, including total cholesterol, LDL-C, triglycerides and blood pressure, whereas HDL-C levels were higher (137). Therefore, long-term caloric restriction appears to be a possible strategy for atherosclerosis prevention. Of particular interest, another study observed that caloric restriction (1,200 kcal/day) for 3 months, combined with physical activity in obese patients with metabolic syndrome led to a surprising decrease of PON1 protein concentration, but increased PON1 activity after weight loss (138). However, in healthy Japanese women, a 2-months intervention of low calorie diet (1,200 kcal/day) was associated with a decrease of PON1 activity (139). It has been hypothesized that this reduction in enzyme activity is an adaptation to reduced LDL-C and HDL-C levels because of a reduced need for antioxidant protection of lipoproteins, but this seems a somewhat far-fetched hypothesis (139). Montefusco et al. studied the effect of a 6-month hypocaloric diet in patients with metabolic syndrome. They demonstrated a reduction in pro-inflammatory cytokine levels and changes in lipoprotein composition, with an increase in triglycerides and apolipoproteins in HDL (140). Interestingly, a positive correlation was observed between CETP levels and cytokine levels, demonstrating a link between lipids and pro-inflammatory cytokines.

Fasting has been practiced for millennia, but only recently studies have shed light on its role in adaptive cellular responses that appear to reduce oxidative damage and inflammation and optimize energy metabolism (141). Intermittent fasting is defined as a period of time, usually from 12 h to 3 weeks, with little or no food intake and abstention from caloric beverages (141).

In a trial of an alternate day fasting regime, with 25% energy intake on fasting days, the intervention reduced body weight, decreased triglyceride levels, and increased LDL particle size, but did not alter LDL-C and HDL-C levels (142). A short-term intervention of the same fasting regime in obese subjects, showed similar results, with decreases in body weight, systolic blood pressure, triglycerides and LDL-C, while HDL-C remained unchanged (143). A comparison of alternate day fasting with a low-fat diet vs. alternate day fasting with high-fat diet in obese subjects showed a decrease of small LDL particles in both groups, while levels of HDL-C and HDL particle distribution remained unchanged (144). In a study of healthy and non-obese subjects, alternate day fasting for more than 6 months showed improved cholesterol, LDL-C, and VLDL levels but had no effect on HDL-C levels (145). Summarized, intermittent fasting appears to have no direct effect on HDL-C levels, but the possible influences of this diet on HDL functionality remain to be investigated.

## Impact of Dietary Intake of Polyphenols on HDL Function

Polyphenols are a large heterogenous family of naturally occurring molecules, which are characterized by the presence of one or more aromatic rings and attached hydroxyl groups (146). More than 8,000 phenolic structures have been reported and most of which are present in plant-based food (146). Dependent on their chemical structure, polyphenols are classified into flavonoids and non-flavonoids (147). Flavonoids are the most numerous of the phenols and are abundant in the entire plant kingdom (148). In recent years, many studies have focused on elucidating the biological activity of polyphenols and polyphenol-enriched foods. While many rodents and *in vitro* studies have been conducted, the available evidence in humans is scarcer. It has been reported that polyphenols exert effects on modulation or prevention of hypertension (149, 150), cardiovascular disease (151, 152), endothelial dysfunction (153), and metabolic syndrome (154). Moreover, recent research has shown that polyphenol intake may also affect HDL composition and functional parameters, such as PON1 activity and cholesterol efflux capacity (155). Therefore, it may be worth considering polyphenols as a dietary supplement to improve HDL functionality. However, further studies are needed to draw firm conclusions.

### Anthocyanin

One group of polyphenols belonging to the flavonoid family are anthocyanins, common water-soluble pigments found in flowers and fruits. Structurally, anthocyanins consist of an anthocyanidin (aglycone) and glycosidically bound sugars (156). Studies have reported that these flavonoids possess antioxidative (157) and anti-microbial activities and also improve the lipid profile of healthy adults (158). Several studies have also demonstrated a preventive effect on diseases, such as CVD and diabetes (156).

In recent years, research has also focused on the bioactivity of this flavonoid subclass in the context of HDL composition and function. In a study cohort of dyslipidemic subjects, anthocyanin supplementation (320 mg anthocyanin capsules/day) for 12 weeks led to an increase of HDL-C by 13.7% with a concomitant elevation of cholesterol efflux capacity to serum (159). Furthermore, anthocyanin supplementation resulted in a decrease of plasma CETP mass and activity, which explains the rise in HDL-C (159). Xu et al. showed that anthocyanin supplementation (80–320 mg/day) improved HDL cholesterol efflux capacity and HDL-C levels (160). The increase of HDL-C upon anthocyanin supplementation in hypercholesterolemic patients has been confirmed in other studies, which also reported improved endothelium-dependent vasodilatation (161) and reduced inflammatory response (162). A 24-week consumption period of anthocyanin increased PON1 activity by 17.4% while enhancing antioxidative capacity and reducing HDL-associated lipid hydroperoxides (163). The study further reported an increase of HDL cholesterol efflux capacity (163). Furthermore, anthocyanin supplementation in a cohort of diabetic patients improved dyslipidemia associated with increased HDL-C and antioxidative capacity of plasma (164). Intake of anthocyanine-rich blueberries over a 6-month period resulted in increased HDL-C, as well as HDL particle number and improved vascular function in overweight and obese subjects (165).

Taken together, the results of these studies appear to provide an explanation for the association between anthocyanin intake, increased HDL functionality, and cardioprotection.

### Quercetin and Green-Tea Polyphenols

The most frequently occurring compound in the family of flavonols is quercetin, occuring in sources including onions, apples, broccoli, bilberries, grapes and green and black tea (166). Mechanistic *in vitro* studies on this flavonoid mostly focused on PON1 and reported an increase of its activity after treatment of hepatocytes (167). Other *in vitro* studies showed that quercetin increased the expression level of SR-BI in HepG2 cells in a concentration- and time-dependent manner (168) and raises ABCA1 mRNA levels and HDL- and apoA-I-mediated cholesterol efflux (169). In rodents, the induction of PON1 expression induced by quercetin was confirmed (170, 171). Feeding quercetin for 4 weeks increased hepatic expression and serum activity of PON1. In line, the ability of HDL to protect against oxidation of LDL was increased (170). However, studies on healthy adults receiving different doses (six capsules with a total of 50–150 mg/day) of supplementary quercetin for 2 weeks did not show any change in PON1 activity, which was argued to be caused by differences in quercetin metabolism between rodents and humans. The quercetin dosages were selected based on the 5-, 10, and 15-fold estimated daily intake of quercetin in Germany (50, 100, and 150 mg) (172).

The subclass of flavanols is mainly composed of the compounds catechin and epicatechin, which are predominantly found in cocoa, grapes, wine and green tea (173). Administration of green tea through drinking water over a period of 6 weeks in diabetic rodents improved HDL functionality by increasing serum PON1 activity and reducing oxidation of apoB-containing lipoproteins (174). In another study, ApoE-deficient mice received extra virgin olive oil (EVOO) enriched with green tea polyphenols for 2 months. A significantly improved PON1 activity and an increase in HDL cholesterol efflux capacity was observed (175). In patients with end-stage renal disease supplementation with green tea extract improved PON1 activity and reduced expression of pro-inflammatory cytokines after hemodialysis (176). In a randomized controlled trial with obese subjects comparing the effects of consuming yerba mate, apple tea, or green tea, a significant increase in PON1 activity was found only in the yerba mate group (177). A recent study in hypercholesterolemic rodents demonstrated that long-term administration of matcha green tea (dosage equivalent to 7.5 cups of tea for human individual) led to lower HDL-C, decreased cholesterol efflux capacity as well as reduced cholesteryl-ester transfer to triglyceride-rich particles. Treatment was associated with increased vascular stiffness and greater susceptibility to the development of atherosclerotic lesions (178). Given these controversial results and the lack of literature on human studies, further research is needed to draw firm conclusions.

### Resveratrol

Resveratrol (3,5,4′-trihydroxy-trans-stilbene) belongs to polyphenols' stilbenoids group, possessing two phenol rings linked to each other by an ethylene bridge. This natural polyphenol has been detected in more than 70 plant species, especially in grapes' skin and seeds, and was found in discrete amounts in red wines and various human foods. Resveratrol is known for its antioxidant and anti-inflammatory properties and for its ability to upregulate endothelial NO synthase (179–181), but resveratrol also affects the lipid metabolism. Specifically, resveratrol induced a statin-like inhibition of HMG-CoA reductase in a hyperlipidemic rodent model, and lowered cholesterol, triglyceride, apoB, and CETP concentrations, accompanied by an increase in plasma apoA-I levels (182, 183). Resveratrol supplementation in apoE-deficient mice revealed similar results, showing increased levels of HDL-C but also elevated plasma PON1 activity (184). In addition, treated animals showed fewer atherosclerotic lesions and less presence of adhesion molecules in atherosclerotic vessels (184). The upregulation of PON1 expression upon resveratrol treatment was further confirmed *in vitro* (185) and *in vivo* (186). Oxidized LDL is present in atherosclerotic lesions, and disease progression is thought to be decelerated by inhibiting oxidation (187, 188). Of particular interest, resveratrol prevented LDL from peroxidation induced by copper- and γ-radiolysis in a dose dependent manner (189). Resveratrol was suggested to interact with radicals to form stable or non-radical compounds (190). The effect of resveratrol on cholesterol homeostasis has also been demonstrated through its effect on apoA-I-mediated cholesterol efflux by upregulating ABCA1 (189). Interestingly, cholesterol uptake by macrophages or endothelial cells was diminished in the presence of resveratrol. Further experiments showed that resveratrol protected Cu-induced oxidation of human HDL3, which was isolated from healthy volunteers, in a dose-dependent manner and preserved its cholesterol efflux capacity (189). Interestingly, in a recent study in patients with type 2 diabetes, 8 weeks of resveratrol supplementation (1,000 mg/day) resulted in increased PON1 activity and decreased serum levels of asymmetric-dimethylarginine, an inhibitor of endothelial NO synthase (191). There are several randomized controlled trials investigating the lipid-lowering effects of resveratrol in humans, but the results are inconsistent. Some studies reported a positive effect of resveratrol on lipid levels (192–196), while others showed no significant impact (197–201). Due to its poor solubility and bioavailability, application of resveratrol is still a major challenge for pharmaceutical industry. Further studies are needed to definitively determine the effect of resveratrol on metrics of HDL-function.

### Curcumin

The polyphenol curcumin, is a well-known and commonly used spice in Middle Eastern and South African cuisine, whose bioactive anti-inflammatory, antioxidant and hepato-protective effects have been investigated in several studies (202–207). Of particular interest is the effect of curcumin on lipid metabolism and the resulting protective effect against atherosclerosis (203, 208). Due to its beneficial properties, curcumin has been suggested as a potential therapeutic to augment HDL functionality (209). *In vitro* experiments examining the effect of curcumin treatment on macrophages revealed a dose-dependent increase in cholesterol efflux through increased expression of ABCA1 and SR-BI mediated by heme oxygenase-1 (210). Interestingly, in a study of hypercholesterolemic rabbits, 6 weeks of curcumin treatment resulted in an increase in HDL-C levels, a decrease in plasma CETP levels, and an increase in antioxidant activity (211).

In a study investigating the potential effect of curcuma on the prevention of atherogenesis in healthy subjects, daily administration of ~20 mg curcumin for a period of 30 days improved plasma lipid profile (212). Specifically, LDL-C and apoB levels decreased, while levels of HDL-C and apoA-I increased. However, in another study of healthy middle-aged subjects receiving a daily dose of 80 mg curcumin, supplementation had no effect on plasma cholesterol levels but reduced plasma triglyceride levels (213). Interestingly, this study revealed an increase in plasma nitric oxide levels, while levels of the soluble intercellular adhesion molecule were decreased after the intervention. In contrast to numerous animal studies that showed a decrease in myeloperoxidase activity after curcumin administration (214–216), an unexpected increase was observed in the human study (213). A recently published systematic review on the effect of nano-curcumin supplementation revealed an overall increase in HDL-C levels (217). Encapsulation of curcumin in nanoformulations has been shown to prolong circulation time and increase its bioavailability and solubility in several *in vitro* studies (218–221) and already has been used in some clinical trials (222–225). However, in summary the published studies on the potential effects of curcumin on HDL functionality are still inconsistent and further studies are needed to draw firm conclusions.

## Conclusion

We now understand that the protective effects of HDL are not reflected by the cholesterol content of the particles, so the quality (composition and functionality) of HDL particles must be evaluated. These properties include HDL mediated cholesterol efflux capacity, antioxidant and anti-inflammatory functions, but also immunmodulating and vasoprotective activities.

The benefit of HDL-C elevation is unclear given the conflicting evidence from pharmacological studies on HDL-C elevation, but an examination of the functional properties of HDL deserves attention. Dietary strategies and certain dietary components have been shown to improve HDL functionality. The strongest evidence for modifying parameters of HDL function is available for the Mediterranean diet. This dietary pattern, especially when enriched with EVOO, has been shown to improve HDL cholesterol efflux capacity, to increase PON1 activity, and to augment antioxidant capacity of HDL. Particularly, the phenolic compounds of EVOO seem to exert these effects on HDL function. Supplementation of other polyphenols, such as anthocyanins, but also antioxidants like lycopene and eicosapentaenoic acid appear to improve HDL functionality, highlighting the need for additional research.

## Author Contributions

JS and GM conceptualized and wrote the manuscript. JS generated the figures. All authors contributed to the article and approved the submitted version.

## Funding

This work was supported by the Austrian Science Fund (FWF) (DOC 31-B26) and the Medical University Graz through the PhD Program Inflammatory Disorders in Pregnancy (DP iDP).

## Conflict of Interest

The authors declare that the research was conducted in the absence of any commercial or financial relationships that could be construed as a potential conflict of interest.

## Publisher's Note

All claims expressed in this article are solely those of the authors and do not necessarily represent those of their affiliated organizations, or those of the publisher, the editors and the reviewers. Any product that may be evaluated in this article, or claim that may be made by its manufacturer, is not guaranteed or endorsed by the publisher.

## Abbreviations

ABCA1: ATP-binding cassette A1
ABCG1: ATP-binding cassette G1
Apo: apolipoprotein
CETP: cholesteryl-ester transfer protein
CHD: coronary heart disease
CVD: cardiovascular disease
DHA: docosahexaenoic acid
EPA: eicosapentaenoic acid
EVOO: extra virgin olive oil
HL: hepatic lipase
LCAT: lecithin-cholesterol acyltransferase
LPS: lipopolysaccharide
NO: nitric oxide
PON1: paraoxonase1
SAA: serum amyloid A
S1P: sphingosine-1-phosphate.
